# Supplementary material for: Treatment strategies for non-responders to oral iron and folic acid treatment in anemic children: A systematic review
Source: PLOS Glob Public Health. 2025 Mar 13;5(3):e0003870. doi: 10.1371/journal.pgph.0003870 (PMC11906079; doi:10.1371/journal.pgph.0003870)
Supplement: S1 Table — (DOCX) [file pgph.0003870.s001.docx]

*S1_Table: Detailed Search strategy*

1. ***Medline***

| ***Concept*** | ***Mesh terms*** | ***Text words*** |
| --- | --- | --- |
| *Anemia* | *Anemia* | *"anemia/epidemiology"[MeSH Major Topic] OR "Anaemia/epidemiology"[MAJR] OR "anemia, iron deficiency/epidemiology"[MeSH Major Topic] OR "Nutritional Status"[MeSH Terms] OR "Government Programs"[MeSH Major Topic] OR "iron, dietary/administration and dosage"[MeSH Terms] OR "Iron/administration and dosage"[MAJR]) OR "Folic Acid/administration and dosage"[MAJR]* |
| *Treatment failure* | *Treatment failure* | *"treatment-refractory"[Tw] OR "refractory"[Tw] OR "treatment resistan*"[Tw] OR "non-responder"[Tw] OR "Treatment failure"[Tw] OR "Therapy failure"[Tw] OR "Failure to respond"[Tw] OR Non-response to treatment[Tw] OR "Drug failure"[Tw] OR "Treatment non-response"[Tw] OR "Treatment nonresponse"[Tw] OR "Unsuccessful treatment"[Tw]* |

*("anemia/epidemiology"[MeSH Major Topic] OR "Anaemia/epidemiology"[Tw] OR "anemia, iron deficiency/epidemiology"[MeSH Major Topic] OR "Nutritional Status"[MeSH Terms] OR "Government Programs"[MeSH Major Topic] OR "iron, dietary/administration and dosage"[MeSH Terms] OR "Iron/administration and dosage"[MAJR] OR "Folic Acid/administration and dosage"[MAJR]) AND ("treatment-refractory"[Tw] OR "refractory"[Tw] OR "treatment resistan*"[Tw] OR "non-responder"[Tw] OR "Treatment failure"[Tw] OR "Therapy failure"[Tw] OR "Failure to respond"[Tw] OR Non-response to treatment[Tw] OR "Drug failure"[Tw] OR "Treatment non-response"[Tw] OR "Treatment nonresponse"[Tw] OR "Unsuccessful treatment"[Tw])*

***Hit results (dated 31/05/2024)***

| ***Query*** | ***Results*** |
| --- | --- |
| *#1 Anemia* | *75,598* |
| *#2 Treatment failure* | [2,32,5*10*](https://pubmed.ncbi.nlm.nih.gov/?term=%28%22anemia%2Fepidemiology%22%5BMeSH+Major+Topic%5D+OR+%22anemia%2C+iron+deficiency%2Fepidemiology%22%5BMeSH+Major+Topic%5D+OR+%22Nutritional+Status%22%5BMeSH+Terms%5D+OR+%22Government+Programs%22%5BMeSH+Major+Topic%5D+OR+%22iron%2C+dietary%2Fadministration+and+dosage%22%5BMeSH+Terms%5D%29+&sort=&size=100) |
| *#1 AND #2* | ***316*** |

1. ***EMBASE***
2. *Anemia*

*(iron or ferric or ferrous or anemi* or anaemi* or transfus*).ti,ab,kw*

***Performed search 1:*** *iron:ti,ab,kw OR ferric:ti,ab,kw OR ferrous:ti,ab,kw OR anemi*:ti,ab,kw OR anaemi*:ti,ab,kw OR transfus*:ti,ab,kw*

***Results:*** *735,609*

1. *Failure to treatment*

*("treatment-refractory", "refractory", "treatment resistan*, "non-responder", "Treatment failure","Therapy failure", "Unsuccessful treatment").ti,ab,kw*

***Performed search 2:*** *'treatment-refractory':ti,ab,kw OR 'refractory':ti,ab,kw OR 'treatment resistan*':ti,ab,kw OR 'non-responder':ti,ab,kw OR 'treatment failure':ti,ab,kw OR 'therapy failure':ti,ab,kw OR 'unsuccessful treatment':ti,ab,kw*

***Results****: 336,641*

*3. Combined search?*

*1 AND 2: 20,703 results*

*1 AND 2 (filters: Humans): 13872 results*

***GOOGLE SCHOLAR***

*"Anemia" AND "Treatment failure"*

*"Anemia" AND "Therapy failure"*

*"Anemia" AND "Failure to respond"*

*"Anemia" AND "Failure of therapy"*

*"Anemia" AND "Non-response to treatment"*

*"Anemia" AND "Drug failure"*

*"Anemia" AND "Treatment non-response"*

*"Anemia" AND "Treatment nonresponse"*

*"Anemia" AND "Unsuccessful treatment"*

*Results: 54*
