# Supplementary material for: Treatment strategies for non-responders to oral iron and folic acid treatment in anemic children: A systematic review
Source: PLOS Glob Public Health. 2025 Mar 13;5(3):e0003870. doi: 10.1371/journal.pgph.0003870 (PMC11906079; doi:10.1371/journal.pgph.0003870)
Supplement: S2 Table — (DOCX) [file pgph.0003870.s002.docx]

**S2_Table:**  List of excluded articles and the reasons for exclusion

|  | **Articles** | **Reason for Exclusion** |
| --- | --- | --- |
| 1 | Gargallo-Puyuelo 2018, Iron Treatment May Be Difficult in Inflammatory Diseases: Inflammatory Bowel Disease as a Paradigm | Review of treatment focused on Inflammatory Bowel Disease (IBD) |
| 2 | Talarico 2021, Iron deficiency anemia refractory to conventional therapy but responsive to feralgine® in a young woman with celiac disease | Genetic disease |
| 3 | Pavord 2017, Management of pregnant anaemic women not responding to oral iron | Review article / guidelines |
| 4 | PérezRoldán 2008, Effect of Helicobacter pylori eradication on iron deficiency anemia of unknown origin | Age >50 years; H. Pylori cases |
| 5 | Asperti 2021, Iron distribution in different tissues of homozygous Mask (msk/msk) mice and the effects of oral iron treatments | Animal studies |
| 6 | Ovilla 2014, Experience of a single Latin-American institution using a colloidal iron oxide coated with a semisynthetic carbohydrate (ferumoxytol) for iron deficiency anemia (IDA) in patients with intolerance or treatment failure with oral iron | Adult population |
| 7 | Johnson-Wimbley 2011, Diagnosis and management of iron deficiency anemia in the 21st century | Review article on mechanisms and different iron formulations |
| 8 | Akarsu 2006, Treatment of Iron Deficiency Anemia with Intravenous Iron Preparations | Children who were given oral iron previously were excluded |
| 9 | Suzuki 2016, Iron deficiency anemia refractory to iron preparations | Japanese article; review on failure to oral iron |
| 10 | Bastida 2021, Sucrosomial Iron Supplementation for the Treatment of Iron Deficiency Anemia in Inflammatory Bowel Disease Patients Refractory to Oral Iron Treatment | Ulcerative colitis and age > 18 years |
| 11 | Yoon 2011, Iron deficiency anemia in childhood | Original article in Korean language |
| 12 | Van Wyck 2009, Large-dose intravenous ferric carboxymaltose injection for iron deficiency anemia in heavy uterine bleeding: a randomized, controlled trial | Women of reproductive age (WRA) |
| 13 | Akin 2014, Responsiveness to parenteral iron therapy in children with oral iron-refractory iron-deficiency anemia | Genetic disease |
| 14 | Mantadakis 2016, Intravenous iron sucrose for children with iron deficiency anemia: a single institution study | Genetic disease |
| 15 | Sourabh 2019, Favorable improvement in haematological parameters in response to oral iron and vitamin C combination in children with Iron Refractory Iron Deficiency Anemia (IRIDA) phenotype | Genetic disease |
| 16 | Powers 2019, The Development of Ironchild: A Web-Based Intervention to Improve Adherence in Children with Iron Deficiency Anemia | Theoretical intervention to improve adherence |
| 17 | Powers 2017, Caregiver-perceived facilitators and barriers to oral iron therapy in young children with iron deficiency anemia | Qualitative study on caregivers’ perception (oral iron failure) |
| 18 | Kotb 2012, The impact of H. pylori eradication on response to oral iron therapy in patients with iron deficiency anemia | Adult population |
| 19 | Tahaineh 2017, Evaluation of factors in a primary care setting which may cause failure to respond to oral iron treatment in iron deficiency anemia patients | Study on factors associated with iron therapy failure |
| 20 | Khuong-Quang 2013, Iron refractory iron deficiency anemia: presentation with hyperferritinemia and response to oral iron therapy | Genetic disease |
