# Supplementary material for: Treatment strategies for non-responders to oral iron and folic acid treatment in anemic children: A systematic review
Source: PLOS Glob Public Health. 2025 Mar 13;5(3):e0003870. doi: 10.1371/journal.pgph.0003870 (PMC11906079; doi:10.1371/journal.pgph.0003870)
Supplement: S3 Table — (DOCX) [file pgph.0003870.s003.docx]

**S3_Table:** Detailed Data Extraction Table

| Study author(s) | Study Title | Name of Data Extractors | Date of Data Extraction | Year in which the study was conducted | Country | Disease condition | Type of study design | Definition of treatment failure | Sample size | Age | Sex | Inclusion | Exclusion | Route of administration | Frequency of administration | Intervention | Duration of follow-up | Outcome | Conclusion |
| --- | --- | --- | --- | --- | --- | --- | --- | --- | --- | --- | --- | --- | --- | --- | --- | --- | --- | --- | --- |
| Powers 2015 | Efficacy and Safety of Intravenous Ferric Carboxymaltose in Children  with Iron Deficiency Anemia Unresponsive to Oral Iron Therapy | Rukman M, Ranadip C | 01-06-2024 | 2014-15 | USA | IDA | Retrospective cohort | Failure of oral iron therapy (not defined) | 87 | 9 months to 20.8 years (median age of 14 years) | B | Children with IDA who failed oral iron therapy and received FCM | Nil | IV | Two doses administered at least 7 days apart | Ferric carboxymaltose (FCM); Single dose | 6 weeks | post-initial infusion with a median hemoglobin increase of 3.3 g/dL (range -1.5 to 9.5 g/dL). Serum ferritin (ng/mL),  Pre (n=80): 5.2 (0.6 to 288.6)  Post (n=60): 115.7 (2.3 to 679.3) | IV FCM in an outpatient setting as one or two short IV infusions was effective in most children and adolescents with IDA refractory to oral iron therapy. |
| Powers 2017 | Intravenous Ferric Carboxymaltose in Children with Iron Deficiency  Anemia Who Respond Poorly to Oral Iron | Rukman M, Ranadip C | 01-06-2024 | 2014-15 | USA | IDA | Retrospective cohort | Taken oral iron therapy for a median of 4 months (IQR, 2-12 months) yet exhibited limited or no increase above their baseline Hgb concentration | 72 | 9 months to 20.8 years (median age of 14 years) | B | Children with IDA who failed oral iron therapy and received FCM | Patients without anemia who received FCM for other indications were excluded from the analysis | IV | Single ot two | Ferric carboxymaltose (FCM); median dose 435 mg | 4 and 12 weeks | Hb g/dL: Pre- 5.4 (5.5-12.2) Post - 12.3 (8.8-16) | FCM administered as a short IV infusion in a diverse group of infants, children, and adolescents with refractory IDA resulted in satisfactory hematologic responses and infrequent adverse effects. |
| Ozsahin 2020 | Intravenous ferric carboxymaltose for iron deficiency anemia  or iron deficiency without anemia after poor response to oral  iron treatment: Benefits and risks in a cohort of 144 children  and adolescents | Rukman M, Ranadip C | 04-06-2024 | 2017-19 | Switzerland | IDA | Retrospective cohort | Failure of oral iron therapy | 144 | 18 mon to 18 years | B | children and adolescents with IDA/ID and inadequate or failed response to oral iron therapy, who received intravenous FCM at |  | IV | Single | Ferumoxytol (FCM) infusion; s 20 mg/kg or 1000 mg for one infusion session. | 6 to 12 weeks | For < 6 years: 85% achieved the target ferritin level ≥ 30 µg/L after a single FCM dose |  |
| Plummer 2013 | Intravenous Low Molecular Weight Iron Dextran in Children With Iron Deficiency  Anemia Unresponsive to Oral Iron | Rukman M, Ranadip C | 13-06-2024 | 2010-12 | USA |  | Observational case series | Children who failed `to respond to or not felt to be candidates for oral iron | 31 | 11 mon to 18 years | B | Children who failed `to respond to or not felt to be candidates for oral iron |  | IV | Single | A total dose infusion of low molecular weight iron dextran (LMWID) administered over 60 minutes in the outpatient;MAX 1000MG |  | Significant Median hemoglobin rise in each of the subgroups- nutritonal iron deficiency, chronic blood loss. Overall: Sixteen (67%) exhibited a complete response and the remaining 33% a partial response | A demonstrable response, as determined by an increase in the Hb concentration andMCVand/or serum ferritin in each of the 24 evaluable patients |
| Crary 2011 | Intravenous Iron Sucrose for Children With Iron Deficiency Failing to  Respond to Oral Iron Therapy | Rukman M, Ranadip C | 01-07-2024 | 2004-09 | USA |  | Record based study | Failing to Respond to Oral Iron Therapy | 13 | 3 months to 18 years (median 5 years) | B | Failing to Respond to Oral Iron Therapy | Kidney diseases | IV | At least one dose | IV sucrose; individual doses ranged from 25 to 500 mg (median 300 mg); median times of dosing 3 |  | Hb increased overall and was superior to oral iron in the children refractory to oral iron or with malabsorption (P < 0.001 and P < 0.04, respectively). |  |
| Sarker 2008 | Causal Relationship of Helicobacter pylori With Iron-Deficiency Anemia or  Failure of Iron Supplementation in Children | Rukman M, Ranadip C | 12-06-2024 | 1998 - 2002 | Bangladesh | Hpylori | RCT | Failing to Respond to Oral Iron Therapy | 260 | 2-5 years |  | IDA |  | Oral |  | 200 Hp-infected children (2-5 years of age) with IDA or ID- 2-week anti-Hp therapy (amoxicillin, clarithromycin, and omeprazole) plus 90-day oral ferrous sulfate (anti-Hp plus iron) 2-week anti-Hp therapy alone 90-day oral iron alone Placebo | 90 days | In all 4 intervention groups, the Hb levels increased significantly. Improvement of iron in children who received combined therapy can be attributed to the effect of iron rather than anti-H pylori therapy | H pylori-infected children receiving iron had significantly less frequent treatment failure compared with those with no iron therapy in correcting IDA on day 90 (11% for anti-H pylori plus iron, 0% for iron alone vs 33% for anti-H pylori and 45% for placebo; p <0.0001). |
| Giovanna Russo et al. | Monitoring oral iron therapy in children with iron deficiency anemia:  an observational, prospective, multicenter study of AIEOP patients | Rukman M, Ranadip C | 01-07-2024 | 2015-2016 | Italy | Iron deficiency anemia (IDA) | Observational, prospective, multicenter study | Lack of normal value restoration of transferrin saturation and Hb levels | 107 | 3 months to 12 years | M = 64, F = 43 | Children aged 3 months to 12 years with Hb < 10 g/dl (≤24 months), Hb < 11 g/dl (>24 months), MCV < 70 (≤24 months); MCV < 74 (>24 months), and transferrin saturation < 15% | Children with conditions affecting iron absorption (e.g., coeliac disease, infections, chronic diseases, thalassemia, etc.), prior IV iron or transfusion | Oral iron (ferrous salts, bis-glycinate iron, liposomal iron) | Once daily | Various oral iron formulations: ferrous gluconate/sulfate (2 or 4 mg/kg), ferric iron salts, bis-glycinate iron, liposomal iron | 24 weeks | Median increase in hemoglobin levels at 2 and 8 weeks, reticulocyte increase at 3 days, gastrointestinal side effects (16% for ferrous salts, 6% for bis-glycinate) | Bis-glycinate and liposomal iron formulations showed a good efficacy/safety profile; ferrous salts had higher Hb increase but more side effects |
