# Supplementary material for: Treatment strategies for non-responders to oral iron and folic acid treatment in anemic children: A systematic review
Source: PLOS Glob Public Health. 2025 Mar 13;5(3):e0003870. doi: 10.1371/journal.pgph.0003870 (PMC11906079; doi:10.1371/journal.pgph.0003870)
Supplement: S4 Table — (b) Summary of Cochrane Risk of Bias (RoB) for Randomized Controlled Trials. (c) JBI Critical Appraisal for Case Series. (DOCX) [file pgph.0003870.s004.docx]

**S4a_Table: Risk of bias assessment**

| Study ID | Selection | | | Comparability | Outcome | | Total |
| --- | --- | --- | --- | --- | --- | --- | --- |
|  | Representativeness of the exposed cohort (*) | Selection: non exposed cohort (*) | Ascertainment of exposure (*) | Comparability of groups on basis of design or analysis (**) | Outcome: assessment(*) | Adequacy of follow-up (*) | 8⋆ |
| Akin 2014 | - | * | * | - | * |  | 3* |
| Powers 2015, 2017 | - | * | * | ** | * |  | 5* |
| Ozsahin 2020 | - | * | * | ** | * |  | 5* |

**S4b_Table. Summary of Cochrane Risk of Bias (RoB)**

| **Domain** | **Details for Sarkar 2008 Study** | **Risk of Bias Judgment** |
| --- | --- | --- |
| **1. Random Sequence Generation** | The study mentions a randomization process using "random permuted block numbers." | Low |
| **2. Allocation Concealment** | Bottles were arranged in sequence corresponding to the randomization chart and were serially numbered. Blinding appears appropriate. | Low |
| **3. Blinding of Participants and Personnel** | The study was double-blind with participants, caregivers, and investigators blinded. | Low |
| **4. Blinding of Outcome Assessment** | Outcome assessors (laboratory-based measures) were likely blinded as there was no mention of blinding concerns affecting results. | Low |
| **5. Incomplete Outcome Data** | Attrition is reported (follow-up data for 47–49 out of 50 participants in each group). There is a balanced dropout across groups. | Low |
| **6. Selective Reporting** | All prespecified outcomes seem to have been reported, including hemoglobin, iron status, and H pylori status. | Low |
| **7. Other Bias** | There are no apparent sources of other bias. | Low |

**S4c_Table. JBI Critical Appraisal for Case Series**

| Study, year | Clear criteria for inclusion | Condition measured in a standard, reliable way | Valid methods for identification of the condition | Consecutive inclusion of participants | Complete inclusion of participants | Clear reporting of demographics | Clear reporting of clinical information | Clear reporting of outcomes | Appropriate statistical analysis | Stratification of cases |
| --- | --- | --- | --- | --- | --- | --- | --- | --- | --- | --- |
| Crary 2011 | Yes | Yes | Yes | Yes | Yes | Yes | Yes | Yes | Not applicable | Yes |
| Plummer 2013 | Yes | Yes | Yes | Yes | Yes | Yes | Yes | Yes | Not applicable | Yes |
| Mantadakis 2016 | Yes | Yes | Yes | Yes | Yes | Yes | Yes | Yes | Not applicable | Yes |
