# Supplementary material for: Treatment strategies for non-responders to oral iron and folic acid treatment in anemic children: A systematic review
Source: PLOS Glob Public Health. 2025 Mar 13;5(3):e0003870. doi: 10.1371/journal.pgph.0003870 (PMC11906079; doi:10.1371/journal.pgph.0003870)
Supplement: S5 Table — (DOCX) [file pgph.0003870.s005.docx]

**S5_Table: List of studies included in the review**

| **Study Number** | **Full Title** | **Authors** | **Year of Publication** |
| --- | --- | --- | --- |
| 1, 2 | *Intravenous Ferric Carboxymaltose in Children with Iron Deficiency Anemia Who Respond Poorly to Oral Iron*  *Efficacy and safety of intravenous ferric carboxymaltose in children with iron deficiency anemia unresponsive to oral iron therapy* | Powers JM, et al. | 2015, 2017 |
| 3 | *Intravenous ferric carboxymaltose for iron deficiency anemia or iron deficiency without anemia after poor response to oral iron treatment: Benefits and risks in a cohort of 144 children and adolescent* | Ozsahin H, et al. | 2020 |
| 4 | *Intravenous low molecular weight iron dextran in children with iron deficiency anemia unresponsive to oral iron* | Plummer ES, et al. | 2013 |
| 5 | *Intravenous iron sucrose for children with iron deficiency failing to respond to oral iron therapy* | Crary SE, et al | 2011 |
| 6 | *Causal Relationship of Helicobacter pylori with Iron-Deficiency Anemia or Failure of Iron Supplementation in Children* | Sarker SA, et al. | 2008 |
| 7 | *Monitoring Oral Iron Therapy in Children with Iron Deficiency Anemia: An Observational Prospective Multicenter Study* | Russo G, et al. | 2020 |
