# Supplementary material for: Treatment strategies for non-responders to oral iron and folic acid treatment in anemic children: A systematic review
Source: PLOS Glob Public Health. 2025 Mar 13;5(3):e0003870. doi: 10.1371/journal.pgph.0003870 (PMC11906079; doi:10.1371/journal.pgph.0003870)
Supplement: Attachment — Submitted filename: Response to reviewers.docx [file pgph.0003870.s006.docx]

Rebuttal Letter for Manuscript PGPH-D-24-00950 (Previously Submitted)

Dear Editor,

Thank you for your time and consideration of my manuscript, "Effective Treatment Strategies for Women and Children Under Five: Addressing Non-Responders to Oral Iron and Folic Acid- A Systematic Review" (PGPH-D-24-00950). I am writing to address the concerns raised and respectfully request the opportunity to resubmit the manuscript for your review.

Addressing Concerns:

I acknowledged that the original literature search concluded on March 1, 2023. To ensure the review reflected the latest research, I conducted a comprehensive update, including all relevant studies published up to May 30, 2024.

The reviewers highlighted the lack of an appraisal of study quality and risk of bias. I incorporated the Newcastle-Ottawa Scale (NOS) to assess the methodological quality of the included studies. The results of this assessment are included in the revised manuscript.

I believe these revisions addressed the concerns raised by the editors and significantly improved the strength and comprehensiveness of the systematic review. I am confident that the updated manuscript met the high standards of your journal.
