# Supplementary material for: Treatment strategies for non-responders to oral iron and folic acid treatment in anemic children: A systematic review
Source: PLOS Glob Public Health. 2025 Mar 13;5(3):e0003870. doi: 10.1371/journal.pgph.0003870 (PMC11906079; doi:10.1371/journal.pgph.0003870)
Supplement: Attachment — Submitted filename: Response to Reviewer - Round 2.docx [file pgph.0003870.s009.docx]

**Reviewer #3:**

We sincerely thank the reviewers for taking the time to provide insightful feedback on our manuscript. Thanks to the editors for the opportunity to address feedback from a second round of review. Below, we provide a point-by-point response to each comment.

1. **Why only include anemic children? It would be relevant to assess non-responsiveness to IFA in both anemic and non-anemic populations for increased generalizability (and iron deficient and replete, as well).**

Response: Thank you for the insightful feedback. The inclusion of only anemic children in this review aligns with the objectives outlined by the Indian Council of Medical Research (ICMR), which initiated this project to develop effective treatment strategies specifically for children with anemia who do not respond to standard oral iron and folic acid (IFA) treatment. The focus on this vulnerable population was mandated by the project's primary goal of addressing gaps in care where standard treatment has proven insufficient.

As registered in the PROSPERO protocol (CRD42024552200), the study population is defined as children between 0 to 59 months of age who are anemic. Including non-anemic children or those with adequate iron stores would have broadened the scope beyond the intended objective, potentially diluting the study's relevance to this high-risk group. This focused approach ensures the study directly addresses the needs of children most at risk, thereby maximizing its impact on national health priorities and informing tailored interventions for refractory anemia.

2. **Further, i think it is worth comment on how anemia was assessed across these eligible studies. Single-drop capillary blood has shown to render highly inaccurate estimations of hemoglobin concentration. Was single-drop or venous blood used, and how was anemia defined?**

Response: Thank you for the insightful comment. We have now added this information to the results section.

Lines 196- 202: The included studies used varying methods to assess anemia, with some explicitly using venous blood samples (e.g., Sarker 2008, Russo 2020) while others did not specify the sampling technique (e.g., Crary 2011). In studies that included measurements of serum ferritin or transferrin saturation (e.g., Powers 2015, Powers 2017, Ozsahin 2020), venous blood sampling was likely used.

Definitions of anemia, even though not clearly stated, were generally based on hemoglobin concentration thresholds, often aligning with WHO criteria, and some studies incorporated additional parameters such as ferritin or transferrin saturation levels to confirm iron deficiency.

3. **It is not clear why the title includes 'effective treatment strategies' as i do not think this can be confirmed from the scope and content of this review.**

Response: The word “Effective” removed as suggested

4. **Was the review registered in any online repository, such as PROSPERO? Were there any deviations from the initial protocol?**

Response: As already mentioned in the methods, the protocol for this review has been officially registered with PROSPERO, the international prospective register of systematic reviews. The registration number is CRD42024552200.

There is no deviation of initial protocol. As suggested by the previous reviewer we are not presenting evidence of women of reproductive age in the same manuscript.

5. **Line 86-93: the scope of the search seems limiting, e.g., use of several very specific key word searches. Did the authors seek help from a librarian or expert in literature searches to execute the search?**

Response: Response to Reviewer Question:

Thank you for enquiring about the scope of the search strategy. We carefully designed our search to ensure a comprehensive inclusion of relevant studies while avoiding excessive specificity that might limit the breadth of results. The search terms were intentionally selected to balance specificity and inclusivity, capturing the key concepts of our research question without overly narrowing the scope.

For example:

The use of MeSH terms like “Anemia/epidemiology” ensured the inclusion of studies relevant to anemia (direct and indirect).

The addition of terms like “treatment-refractory,” “non-responder,” and “treatment-resistant” allowed us to target studies focused on non-responders to iron and folic acid (IFA) treatment, which is the primary focus of our review.

While adding more specific search blocks can seem like a way to increase precision, it can inadvertently act as a filter, excluding studies that do not precisely match the specified combinations of terms. In the methods section of the manuscript, only the guiding principles of our search strategy is given. The supplementary table provides a comprehensive list of all possible synonyms and keyword combinations used within each search block. The search was not confined to specific populations or regions, ensuring global relevance.

While we did not specifically consult a librarian, the search strategy was developed collaboratively by a team of experienced researchers familiar with systematic review methodologies (trained in literature review by QMed foundation and were involved in WHO Guideline Development Evidence Synthesis). Furthermore, the search strategy underwent several rounds of refinement and testing based on known literature to see if studies were missed during initial search.

6. **Abstract: Suggest to revise first sentence as it implies the only consequence of IFA deficiency is anemia; when in fact, there are numerous and some of which are more important than anemia.**

**Suggest to replace the word 'suffer' with more objective text.**

**Please clarify what is meant by 'standard IFA treatment'.**

**Authors mention case series (do they mean case studies) or is clarification required here? No need to capitalize the word 'intravenous'.**

Response: Revised as follows:

Lines 23- 27: Iron and folic acid (IFA) are crucial essential nutrients, with deficiencies associated not only with anemia but also with other significant health consequences, including impaired cognitive development, increased susceptibility to infections, and adverse pregnancy outcomes leading to anemia, a global health problem affecting many children.

The term "case series" refers to a specific study design where a group of patients with a shared condition or treatment is described to evaluate outcomes. This is different from "case studies," which typically focus on detailed observations of a single patient. These designs were accurately described by the original authors of the included studies, and our review adheres to their terminology to maintain consistency of the source data.

Changed the word suffer: Despite the widespread use of IFA for management, a notable percentage of individuals failed to respond resulting in persistent anemia.

7. **Please add more detail to tool(s) used assess the quality of bias?**

Response: Thank you for the comment.

We have added a section on Assessment of Quality

Lines 123 – 129: The risk of bias in the included studies was assessed using the Newcastle–Ottawa Scale (NOS) for observational studies. The NOS scores were categorized as high risk of bias (total score of 0–2), moderate risk of bias (total score of 3–5), and low risk of bias (total score of 6–8). For RCTs, the Cochrane Risk of Bias (ROB) tool was utilized to provide a more suitable quality assessment. Case series were assessed using the JBI tool for case series to determine their quality. Two review authors (R M. and R.C.) independently assessed the risk of bias.

Table 4a, 4b and 4c has summarized the quality of evidence.

8. **Line 107: with what software?**

Response: Modified to mention covidence software.

9. **Line 78: was this assessed within a specific timeframe? when was 'non-response' assessed?**

Response: In the methods, we have defined that Non-responders were defined as anemic individuals who did not recover after the standard IFA regimen or as defined by the study authors. The details are in the study details. However as per your suggestion we have added the following sentence to the results section. Thank you for giving suggestion to increase the clarity of manuscript.

Lines 151 – 154: The included studies varied in their definitions of non-response to IFA treatment. Generally, non-response was described as the failure to achieve a clinically meaningful increase in hemoglobin levels after a standard treatment period, which typically ranged from 8 to 12 weeks. Specific definitions and follow-up durations for each study are detailed in Supplementary Table 3.

10. **Adherence to the IFA is obviously a major potential confounder to the non-response outcome. Please expand upon how this was assessed across the different studies and if authors feel this was comprehensive enough to tease out the effect from these two variables.**

Response: Thank you for the suggestion. We have added the following lines to the results section accordingly.

Lines 192 - 195: In the study by Sarkar et al., iron supplementation was monitored for 90 days, with adherence ensured through supervised iron administration. Similarly, Russo et al. (2020) recorded adherence by engaging parents during follow-up visits to assess compliance and report any side effects. However, other studies included in the review did not systematically assess compliance.

**Reviewer #4**

**Many thanks to the Editor for the opportunity to review this paper. The topic is relevant, and anemia is a growing concern for the vulnerable groups, especially in LMICs. Overall, the paper is well written but i have minor concerns that will be helpful to the authors.**

We sincerely thank the reviewers for taking the time to provide insightful feedback on our manuscript. Thanks to the editors for the opportunity to address feedback from a second round of review. Below, we provide a point-by-point response to each comment.

11. **The search terms did not include 'Children', or nutritional anaemia, which are part of the topic. Like, refractory nutritional anaemia in children, to align with research topic and study population. This will help provide more papers to analyze from.**

We appreciate your emphasis on aligning search terms with the study topic and population. However, our search strategy was designed to be broad and inclusive, capturing studies across a wide spectrum.

We employed broad MeSH terms such as "Anemia"[MAJR], which inherently include subheadings and related concepts under its hierarchy, including various types of anemia (e.g., nutritional anemia, and other causes of anemia as well).

- Terms like "Nutritional Status" were connected with "Anemia" using the Boolean operator OR, allowing for studies addressing nutritional aspects of anemia to be included.

- Due to the limited number of studies, we made the search to include all age groups. We then applied inclusion criteria to focus on studies involving children, which was necessary due to the limited number of child-specific studies identified in the initial search.

- The search strategy was developed in collaboration with literature review experts coordinated by the Indian Council of Medical Research (ICMR).

We hope this clarifies our approach. Thank you for raising this important point.

**MINOR:**

1. **There needs to be a review for typos or grammar as seen in line 64, 84, and elsewhere in the paper.**

Response: Thank you. We have now done an external proof reading for the entire manuscript. Apologies for the typos.

2. **In line 59, Oral IFA need to be explicitly qualified. Is it supplementation?**

Response: Thank you for pointing this out. Yes, this was supplementation. We have now modified.

3. **In line 122, JBI should be defined.**

Response: Thank you we have now defined JBI as Joanna Briggs Institute tool.

**RESULTS:**

12. **IV sucrose Vs IV FCM, it will be useful to highlight how the two patient groups differ from the various studies-**

Lines 234- 237: Overall, IV sucrose and IV FCM were both effective in addressing anemia refractory to oral iron. IV sucrose was administered to younger children and required multiple dosing sessions, primarily addressing malabsorption-related anemia. In contrast, IV FCM was predominantly used in older children and adolescents.

13. **The too many exclusions, might be due to search terms not been specific and align to topic.**

Response: This systematic review was commissioned by the Indian Council of Medical Research (ICMR) as part of an initiative to generate evidence for policy decisions regarding the management of anemia in non-responders to oral iron therapy. The research question and scope were carefully designed to align with this objective, focusing on treatment strategies specifically relevant to this vulnerable group.

Search Strategy and Specificity: The search terms were purposefully broad to ensure the inclusion of all potentially relevant studies and avoid missing critical evidence. While this resulted in an initial pool of studies with varying relevance, rigorous screening and exclusion criteria were applied to identify studies that directly addressed the research question. The exclusions were primarily due to studies not aligning with the review’s scope (e.g., focusing on genetic causes of anemia, specific adult populations, or unrelated interventions).

Several criteria for study inclusion and exclusion were developed and refined based on input from three prior rounds of peer review. These suggestions were incorporated to improve the homogeneity of the included studies and ensure the findings are robust and applicable for policymaking. Studies involving genetic or chronic conditions (e.g., thalassemia, chronic kidney disease) were excluded as they require specialized management beyond the scope of nutritional anemia.

**DISCUSSION:**

14. **line 212 does not make a sense or is incomplete.**

Response: Thank you for the suggestion to improve the concept clarity. We have added the following sentence accordingly.

Lines 227 – 229: The effectiveness varied depending on the severity of anemia and patient characteristics. These findings underscore the importance of exploring tailored approaches to address non-responsiveness to oral iron.
