# Supplementary material for: Treatment strategies for non-responders to oral iron and folic acid treatment in anemic children: A systematic review
Source: PLOS Glob Public Health. 2025 Mar 13;5(3):e0003870. doi: 10.1371/journal.pgph.0003870 (PMC11906079; doi:10.1371/journal.pgph.0003870)
Supplement: Attachment — Submitted filename: Response to Reviewer comments.docx [file pgph.0003870.s008.docx]

# Additional Editor Comments

The authors have dealt with an important topic of non-response to oral iron. However, there are methodological weaknesses in the review and the scope of the topic is not clear. Considering the burden of nutritional anemia, the topic should be restricted to nutritional anemia and exclude other studies related to genetic disorders.

Following are few comments and suggestions:

1. *Title: The title should mention clearly that the manuscript describes evidence on non-responders to anemia. Presently the title is broken into three parts and reads a bit awkward.*

**Response**: Thank you for the suggestion. Revised the title to be more specific. “Effective Treatment Strategies for Non-Responders to Iron and Folic Acid (IFA) Treatment in Anemic Women and Children Under Five: A Systematic Review”

2. *Abstract: The search strategy mentioned in the abstract is different from what is mentioned in the methods section. Abstract mentions Cochrane database which is not mentioned in the methods. Also, the start date for search is not clear in the methods. The abstract should be in structured format.*

**Response**: Thank you for the comment. We have revised the same as below:

The electronic databases used for the search included Medline, Cochrane, Google Scholar and Embase, covering the period from January 01, 2000, to dated 31 May 2024

Introduction:

3. *The description should specify the focus on nutritional anemia as there are other etiologies of anemia which may or may not benefit from IFA supplementation. What is the definition of non-responder to IFA in WRA and children? Not clear. Line 57-58 – one of major reason for non-response to IFA could be other etiologies of anemia such as hemoglobinopathies. In this case, establishing the etiology for non-response would be more important. The authors have not elaborated on this aspect.*

**Response**: Addressed as per the suggestions and modified the entire introduction section accordingly.

Methods:

4. *- Details regarding the type of reference software used not mentioned.*

**Response**: Added (line 109)

5. *The methods section does not describe the population covered, type of studies, inclusion exclusion criteria for studies to be included. Also it is not clear whether studies related to non-responders in other disease populations with anemia such as chronic renal failure, inflammatory bowel disease, thalassemia were to be excluded from the manuscript.*

**Response**: Revised as per suggestion (Page 3 – 4).

6. *-New Castle Ottawa scale – This scale can be used for cohort studies. However, for RCTs the authors should have used ROB or other relevant tool. The authors have not assessed the quality of case series although it can be assessed through tools such as JBI tool (*[*https://jbi.global/sites/default/files/2020-07/Checklist_for_Case_Series_0.pdf*](https://jbi.global/sites/default/files/2020-07/Checklist_for_Case_Series_0.pdf)*).*

**Response**. Thank you for this pointing this out. We have now revised the methods based on your suggestion and added Risk of Bias assessment by Cochrane Risk of Bias for RCT and JBI for case series (Supplementary File Table 2a, 2b and 2c).

7. *It is not clear why the authors have not considered reviews or pooled analysis data in this review as there is limited evidence in terms of original studies. E.g. Okam MM, Koch TA, Tran MH. Iron deficiency anemia treatment response to oral iron therapy: a pooled analysis of five randomized controlled trials. Haematologica. 2016 Jan;101(1):e6-7. doi: 10.3324/haematol.2015.129114. This is a very relevant paper which has not been included.*

**Response**: As per the collective suggestion, we have refined our methodology to include only studies involving children. The systematic review was commissioned by the Indian Council of Medical Research, as part of a series of reviews which included women (who failed to respond to oral iron therapy) and children as the population of interest. However, many of those studies are not exclusively on those who failed to respond to oral iron therapy, even though the selected sample includes those.

The study by Okam MM et al. included RCTs comparing alternative treatment regimens with oral iron but did not exclusively focus on non-responders to oral iron. As we have removed studies involving women of reproductive age and pregnant women to better align with the objectives of this review, we hope this revision clarifies the scope and refines the focus of the systematic review. Thank you for the valuable suggestion.

Results

8. *Table 1: It combines many variables into one column. Suggest keeping them separate for better clarity. Definition of non-response in each of these studies should be included in the table. - The authors have combined results from all age groups which should be presented separately. - Details of how many studies in women and children should be included in the description. The search results are questionable – The authors have included about 7-8 studies in pediatric population. However, only couple of studies have been included in women of reproductive age and no studies in pregnant women have been included.*

**Response**: The table has been revised as per suggestion to include more details.

The systematic review was commissioned by the Indian Council of Medical Research, as part of a series of reviews which included women (who failed to respond to oral iron therapy) and children as the population of interest. However, many of those studies are not exclusively on those who failed to respond to oral iron therapy. We have now specifically aligned the review to include studies on children under 18 years.

9. *Case series on IRIDA are included in the results. However, IRIDA is a rare genetic disorder of iron which does not contribute to major burden of anemia. Ideally, these should have been excluded. It is not clear, why the authors have included a study on celiac disease if other causes of anemia like chronic renal disease are excluded. Also, was this a study in WRA or children?*

**Response***:* Excluded as per revised methods now.

Discussion and conclusion

10. *- The authors have not elaborated on the major reasons for non-responders to oral iron in the setting of nutritional anemia which is at the heart of this topic. - Non-compliance to oral iron could be a factor responsible for non-response. The discussion does not cover this. Non-compliance to oral iron is a major public health issue.*

**Response**: Thank you for the comment. Modified the discussion as per the suggestion.

[Lines 215 to 218] One of the major reasons for non-responsiveness to oral iron therapy in the context of nutritional anemia is the poor adherence to treatment. Non-compliance is a widespread issue, particularly in low-resource settings, where factors such as gastrointestinal side effects, the unpleasant taste of iron supplements, and low patient follow-up lead to reduced effectiveness.

11. *- The authors need to elaborate on why in some studies the participants responded to oral iron after initial failure – was it because of high dose or presence of ascorbic acid?*

**Response**: In the currently included studies, Sarker et al. (2008), addressed the underlying infection (H. pylori). However, this did not improve iron absorption. In Giovanna Russo et al. (2015-2016), switching to more bioavailable and better-tolerated forms of iron (like bis-glycinate and liposomal iron) likely contributed to improved outcomes in children who had initially failed to respond to traditional ferrous salt formulations. [Lines 159 to 163]

12. *- Finally the authors have discussed about the need for precision medicines which is a generic truth. The purpose of this review is to collate the available evidence to provide consensus.*

**Response**: Removed as per suggestion.

13. *The authors have not discussed about the best available evidence in specific populations like WRA and children.*

**Response**: Thank you for the comment. The main goal of this review was to collate existing evidence and provide a consensus on effective treatment strategies, particularly for those who fail to respond to oral iron.

14. *The authors do not describe limitations of their review.*

**Response**: Added as per suggestion [Lines 230 to 235]

# Reviewer #1

This is a very important study that systematically identifies non-responders to IFA among high-risk populations in society, and effective treatment regimen for the non-responders. It also serves as the basis for future studies.

Abstract:

15. *It will be more appropriate if the abstract is grouped or differentiated into background, methods, results, and conclusion*

**Response**: Thank you for the suggestion. Revised accordingly.

*Introduction:*

16. *The Introduction gives the background information about non-responders to IFA treatment. Is IFA used for the treatment of all categories of anaemia (mild, moderate, severe)? Hence, the authors should tell the IFA regimen for the type of anaemia in this section.*

**Response:** Thank you for the suggestion. Revised accordingly [Lines 68 to 74]

*Methods:*

*Study selection and eligibility criteria*

17. *Line 87, could the authors mention the name of the reference management software program for reproducibility purposes?*

**Response**: Added as per suggestion [Line 135]

*Assessment of quality*

18. *From line 113, why did the authors combine published articles of qualitative nature (case series, case reports) and that of quantitative strand (RCT, cross-sectional) in this systematic review?*

**Response:** Thank you for your comment. We included case series and case reports due to the limited available evidence on non-responders to oral iron treatment. Given the novelty of this topic and the scarcity of high-quality randomized controlled trials (RCTs), we aimed to provide a comprehensive overview by including all forms of available evidence, as this systematic review represents the first attempt to consolidate data on this specific issue.

However, upon further consideration and in response to your feedback, we have decided to remove case reports from the analysis. We have retained some case series because, in the absence of extensive RCTs, they can provide valuable insights and serve as a basis for future research. As this review is a starting point, we hope it will encourage further investigation, including higher-quality studies such as RCTs or cohort studies. We believe that even qualitative studies, such as case series, can contribute important preliminary data in emerging fields and help guide the design of future, more robust studies.

*Discussion:*

19. *The discussion of the treatment regimen for non-responders to IFA in developing countries should be enhanced since the authors mentioned that anaemia is very prevalent in developing countries.*

**Response**: Thank you for highlighting this important point.

We acknowledge that anemia is a significant public health issue, particularly in developing countries where its prevalence and impact are more severe. However, we included studies from countries such as Italy, the USA, and Switzerland in this review because they provided valuable insights into the management of non-responders to oral iron therapy, which was the focus of our analysis. That said, we agree that more studies focusing on non-responders in developing countries are urgently needed. We have emphasized this point in the revised discussion and limitations sections, noting the need for further research focusing on non-responders to iron therapy in low- and middle-income countries to ensure that findings are relevant and applicable to the regions most affected by anemia.

“Further research is needed focusing on non-responders to iron therapy in low- and middle-income countries to ensure that findings are relevant and applicable to the regions most affected by anemia.” [Lines 283 to 285]

# Reviewer #2

The reviewer appreciates the authors for working on an important public health topic ‘anemia’. However, the manuscript suffers from major methodological inadequacies and weaknesses, that render it unsuitable for publication.

20. *Title: The first half of the title is vague. The term anemia is not reflected in the title.*

*Methods: Why was the search restricted to two search engines? RCTs have been included in the review but clinical trials.gov was not searched*.

**Response**: Thank you for the comment. We addressed the same now.

Regarding Clinical trials, we have done a search now and retrieved no additional trials (till May 31, 2024), based on the 24 results obtained.

21. *A systematic review aims to collect selective published literature on a specific condition to arrive at the direction for the management of a condition. Although anemia is chosen, a well-defined selection criterion for the type of anemia, its severity, and the study setting, type of iron, minimum sample size (clinical or population) would have improved the quality of the selected studies and minimized heterogeneity. Thus, poor selection criteria of the studies have contributed to vague findings.*

*For instance, how can a ‘unique scenario” as described by the authors, a genetic disorder form one case study be compiled together with population-based studies with over 400 samples or RCTs?*

*A well-defined selection criterion would have solved many of the methodological weaknesses.*

**Response:** Thank you for the comment. As per the suggestion from the editor we have now removed the genetic studies. Also revised the method section to reduce heterogeneity.

22. *The quality assessment tool assesses the quality of non-randomized studies. However, the selection included RCTs.*

**Response**: We have revised the Quality assessment tool now to do RCT – Cochrane RoB, for case series JBI tool and NOS for cohort. Details in the supplementary table 2a, b and c.

23. *Table 1: Hb improved from 20-70% is vague. Anemia prevalence decreased or hemoglobin status improved among certain percent etc.*

**Response**: As the inclusion was explicitly IRIDA, we have now removed studies that have included exclusively subjects with genetic causes.

24. *Table 1: How can treatment for mild anemia be compared with a genetic condition or infectious condition?*

**Response**: Removed as genetic studies are excluded now.

25. *Anemia is a public health problem in developing countries, but studies from Italy, USA and Switzerland have been used for comparison.*

**Response:** Thank you for highlighting this important point. We acknowledge that anemia is a significant public health issue, particularly in developing countries where its prevalence and impact are more severe. However, we included studies from countries such as Italy, the USA, and Switzerland in this review because they provided valuable insights into the management of non-responders to oral iron therapy, which was the focus of our analysis. That said, we agree that more studies focusing on non-responders in developing countries are urgently needed. We have emphasized this point in the revised discussion and limitations sections, noting the need for further research focusing on non-responders to iron therapy in low- and middle-income countries to ensure that findings are relevant and applicable to the regions most affected by anemia.

“Further research is needed focusing on non-responders to iron therapy in low- and middle-income countries to ensure that findings are relevant and applicable to the regions most affected by anemia.” [Lines 283 to 285]

26. *The findings of the SR do not contribute to any novel findings: Iron with ascorbate, IV management, or blood transfusion are previously proven strategies.*

**Response:** Yes, indeed. This review has been commissioned by Indian Council of Medical research as a series of reviews for generating evidence related to anemia management in vulnerable groups. Current review tries to bring evidence together for management strategies for failure to respond to oral iron.
